# Hysteroembryoscopy and hysteroscopic uterine evacuation of early pregnancy loss: A feasible procedure in selected cases

**DOI:** 10.52054/FVVO.14.2.020

**Published:** 2022-07-01

**Authors:** U Catena, S D’Ippolito, F Campolo, G Dinoi, A Lanzone, G Scambia

**Affiliations:** Dipartimento di Scienze della Salute della Donna, del Bambino e di Sanità Pubblica, Fondazione Policlinico Universitario A. Gemelli IRCCS, U.O.C. di Ginecologia Oncologica, Rome, Italy; Dipartimento di Scienze della Salute della Donna, del Bambino e di Sanità Pubblica, Fondazione Policlinico Universitario A. Gemelli IRCCS, U.O.C. di Ostetricia e Patologia Ostetrica, Rome, Italy; Università Cattolica del Sacro Cuore, Rome, Italy

## Abstract

Hysteroscopic uterine evacuation of early pregnancy loss using tissue removal devices seems to be a safe and feasible procedure in selected cases. The hysteroscopic approach allows the precise localisation of the gestational sac inside the uterine cavity. The endoscopic approach allows one to perform hysteroembryoscopy before uterine evacuation and this technique appears to be more accurate than dilatation & curettage for fetal chromosome karyotyping, with lower maternal cell contamination. This “under vision” procedure may reduce retained products of conception rates and risk of intrauterine adhesions formation.

## Introduction

Hysteroembryoscopy was first described in 2003, where the procedure was performed under general anaesthesia before dilatation & curettage (D&C) for the treatment of early pregnancy loss (EPL) (Ferro et al. 2003) . EPL is defined as “intrauterine pregnancy with either an empty gestational sac or a gestational sac containing an embryo/fetus without fetal heart activity within 12 weeks of gestation“ (ACOG Practice Bulletin No. 200, 2018). To date, the terms EPL, miscarriage, spontaneous abortion, anembryonic gestation, embryonic or fetal death are used interchangeably.

To perform hysteroembryoscopy, a 4.3 mm hysteroscope (Karl Storz GmbH, Tuttlingen, Germany) is gently introduced into the uterine cavity without cervical dilatation and using normal saline solution as the distension medium. The gestational sac (GS) is visualised, and using a 5 Fr biopsy spoon forceps (Karl Storz GmbH, Tuttlingen, Germany) and a small hole is made in the GS wall. The camera is then gradually introduced into the extracelomic and amniotic cavities which allows complete visualisation of the demised embryo (Abdala et al., 2010). Direct chorion and embryo biopsies are taken before the suction curettage. It has been shown that the detection of fetal chromosomes obtained from direct hysteroembryoscopic biopsies, resulted in more accurate karyotyping with lower maternal cell contamination, than those obtained from curettage material (Cholkeri-Singh et al., 2020). Furthermore, hysteroscopic evacuation of EPL using a 26 Fr monopolar resectoscope has also been shown to be an efficient and safe alternative approach (De Codt et al., 2020). The use of tissue removal devices (TRD) in case of retained products of conception (RPOC) has been widely described and associated with lower risk of intrauterine adhesions (IUAs) formation (Hamerlynck et al., 2016).

The “under vision” technique was proposed for the first time in 2021 (Weinberg et al., 2021) . To treat EPL, they performed a prospective pilot study of 10 patients, which found the intervention safe and feasible in selected cases.

Currently, standard management of EPL includes expectant management, medical treatment using prostaglandin analogues or, alternatively, surgical evacuation by dilatation and curettage (D&C). Expectant management shows a success rate ranging from 25 to 76 % (Jurkovic et al.,1998; Luise et al., 2002). The main limit of this approach include the unpredictability of duration until the resolution of EPL. Medical management is based on prostaglandin analogues showing excellent safety and toxicity profiles. The most commonly used analogue is misoprostoland the success rate of this approach may vary significantly, from 25 to 86%(Neilson et al., 2010; Neilson et al., 2006; Zhang et al., 2005). Surgical treatment is an effective routine and safe procedure based on the tissue removal inside the uterus. However, surgery may increase the risk of complications including cervical trauma as well as bleeding, infection, uterine perforation, RPOC and IUAs formation, possibly due to the "blindness" of the procedure.

## Case report

We describe the case of a 37-year-old woman referred to our department for an asymptomatic EPL based on ultrasonographic findings of fetal demise. We obtained the patient’s written consent to present her case anonymously.

She suffered from primary infertility, and had experienced eight previous failed in-vitro fertilisation (IVF) attempts with 7 years of infertility. Six months prior to the miscarriage, she has been diagnosed with a T shaped uterus and underwent metroplasty with resection of lateral fibromuscular tissue using a 15 Fr bipolar miniresectoscope (Karl Storz GmbH, Tuttlingen, Germany) in our digital hysteroscopy clinic (Catena et al., 2021). Five months after the procedure, she conceived spontaneously and after 7 weeks of amenorrhoea, underwent an ultrasound examination. A monochorionic diamniotic twin pregnancy with two viable embryos was diagnosed.pregnancy with two viable embryos was diagnosed. Beta hCG level was 7532 mIU/mL.

During the 9th week of gestation, a further ultrasound examination revealed a diagnosis of missed miscarriage. Crown-rump lengths (CRL) measurement were 4.4 mm and 3.9 mm respectively and she was referred to the Pregnancy Loss Clinic. After offering her expectant management for 7 days, according to international NICE guidelines (National Institute for Health and Clinical Excellence, 2021), medical treatment with prostaglandin analogue (misoprostol 800 mcg given vaginally on two occasions after 5 days) was offered with informed patient consent. After two administrations of misoprostol, a follow up ultrasound examination showed no response to medical management and retained products in-situ. Considering the patients previous gynaecological history including recent hysteroscopic metroplasty, it was felt a hysteroscopic uterine evacuation was an appropriate next option, to which the patient consented to after informed counselling.

**Figure 1:**
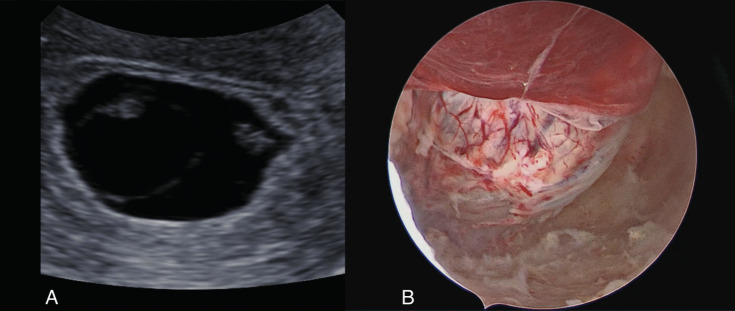
Preoperative images of the missed miscarriage: A) Preoperative ultrasound image; B) Hysteroscopic vision of the GS.

**Figure 2:**
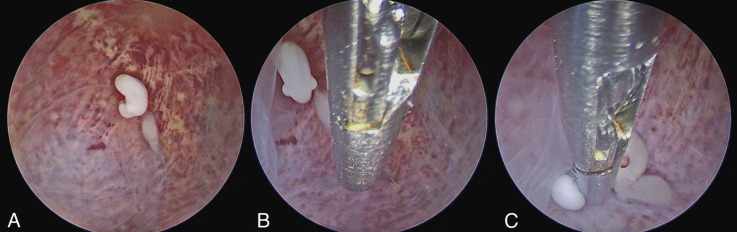
Hysteroembryoscopy: A) Embryo visualisation; B) Introduction of the 5Fr grasping forceps in the operative channel of the hysteroscope; C) Hysteroembryoscopic biopsy with grasping forceps.

**Figure 3:**
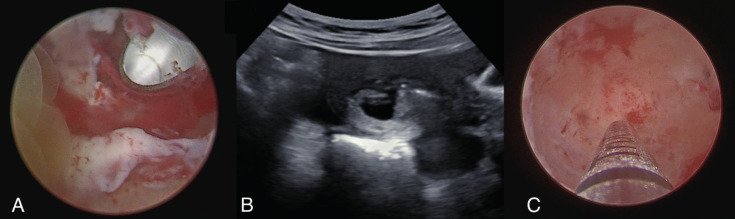
Hysteroscopic Uterine Evacuation with TRD: A) Endouterine Morcellation with Dense Tissue Blade; B) Transabdominal Ultrasound Guidance during the procedure; C) Hysteroscopic view at the end of the procedure.

The patient underwent the hysteroscopic procedure in an outpatient setting under conscious sedation (Level 3(b) pain management, as achieved with i.v. midazolam 10 milligrams and fentanyl 100 micrograms) (Carugno et al., 2021). Before the procedure, a further transvaginal ultrasound examination confirmed the persistence of missed monochorionic diamniotic twin pregnancy. During the examination, one of the two embryos showed an inhomogeneous pattern, likely due to physiological evolutive phenomena (Figure 1A). Hysteroscopy was performed using a vaginoscopic approach with a 5-mm hysteroscope (Karl Storz GmbH, Tuttlingen, Germany). Saline solution was used as distension medium. The uterine cavity was occupied by a gestational sac placed on the right posterio-lateral uterine wall (Figure 1B). The gestational sac was incised with 5Fr scissors inserted in the operative channel of the 5-mm hysteroscope (Karl Storz GmbH, Tuttlingen, Germany). Hysteroembryoscopy was performed with visualisation of one single embryo (Figure 2). Consistently with the ultrasonographic finding, the second embryo, localised in the lower part of the gestational sac was not found. A 5 Fr grasping forceps (Karl Storz GmbH, Tuttlingen, Germany) was used to grab and detach the embryo. After hysteroembryoscopy, we directly inserted the TruClear Elite Mini (diameter 6.25 mm) TRD (Medtronic, Minneapolis, MN), without any dilatation of the cervical canal. With the dense tissue blade (Medtronic, Minneapolis, MN), the uterine cavity was completely evacuated under endoscopic vision and under transabdominal ultrasound guidance (Figure 3). No complications were documented, and patient was discharged 3 hours after the procedure. The average estimated blood loss was 20mL.

The removed tissue was sent for histopathological examination and the fresh specimen was evaluated by an expert pathologist in order to identify the embryo/fetal component. The criteria for a macroscopic identification of the embryo/fetal component required: a more consistent pattern, a translucent grey colour and the embryo/fetal shape. The remaining tissue underwent histological examination. The miscarriage specimens were fixed in 10% buffered formaldehyde for 20–24 hours, embedded in paraffin and stained with hematoxylin- eosin for histological analysis. The definite analysis confirmed missed miscarriage.

## Discussion

Standard surgical management of EPL includes evacuation by D&C. However, it is a blind procedure, which may increase the risk of complications including cervical trauma, bleeding, infection, uterine perforation, RPOC and formation of IUAs (Hooker et al., 2014). In order to preserve future fertility, optimal management of EPL requires complete evacuation of the uterine cavity with minimal endometrial damage. Hysteroscopic evacuation has the potential to achieve this with the advantage it is performed under direct vision (De Codt et al., 2020). Therefore, it may reduce the associated complications with D&C, especially the concern regarding uterine perforation, endometrial trauma, potential IUAs formation and sub-fertility (Vitale et al., 2021).

Hysteroscopic management of RPOC was first described in 1997 (Goldenberg et al., 1997) and this generated numerous papers describing potential clinical settings for the hysteroscopic treatment of RPOC, either in an office setting using hysteroscopic grasper forceps or in a conventional operative room setting using a 26Fr resectoscope (Mohr-Sasson et al., 2022; Golan et al., 2011; Smorgick et al., 2014). More recently, the use of TRD have been proposed to treat endometrial polyps, fibroids and also RPOC (Shazly et al., 2016; Hamerlynck et al., 2016).

TRD has shown to reduce operating time, by simultaneously cutting and suctioning tissue fragments, avoiding the need for multiple removal and reinsertions of the device into the uterine cavity as well as reducing the volume of distension media required to complete the procedure compared to using standard resectoscope (Franchini et al., 2021).

In cases of RPOC, TRD is a faster alternative than loop resection (Hamerlynck et al., 2016). On the basis of this evidence, hysteroscopic treatment was proposed as an option for primary management of EPL. A large case series was recently published using 26 Fr monopolar resectoscope to treat EPL under vision (De Codt et al., 2020), and found it to be an efficient and safe procedure. Weinberg et al. (2021) proposed the use of TRD to primarily treat EPL as a safe and feasible procedure in selected cases (Weinberg et al., 2021). The use of cross- linked hyaluronic acid gel should be considered to prevent IUAs formation after complete evacuation of the uterine cavity (Vitale et al., 2022).

Hysteroscopic treatment of EPL has several limitations compared to D&C, in particularly, the risk of potential fluid overload syndrome and the higher equipment costs. Therefore, the use of hysteroscopy for the treatment of EPL should probably be reserved for selected cases, such as patients with recent uterine surgery, uterine abnormalities or recurrent pregnancy losses. In fact, removing tissue through direct endoscopic visualisation may minimise endometrial damage, decrease the potential trauma to the uterine walls, reduce the risk of RPOC and uterine perforation. Moreover, performing hysteroembryoscopy endoscopically before uterine evacuation seems to be more accurate than D&C for fetal chromosome karyotyping, with lower maternal cell contamination (Cholkeri-Singh et al., 2020).

The main limitation of this paper is that it is a pilot case to assess feasibility and safety of the procedure and it does not allow us to draw any conclusions regarding the possibility to reduce complications or IUAs formation rate after the procedure. Our current experience suggests that hysteroscopic evacuation of the uterine cavity using TRD is an achievable and safe procedure in EPL. By performing the procedure under direct visualisation of the uterine cavity and precisely focusing on the implantation site, we may reduce the risk of RPOC rate as well as the risk of IUAs formation, comparing to the standard surgical or medical treatments used today.

The use of the Truclear Elite Mini could enable this procedure to be performed in an outpatient setting with the patient awake . While this option requires further research, this approach would have to consider the potential emotional and psychological impact for the patient. Further studies are needed to assess the possible benefits of this method and to refine the indications and surgical technique.

## Conclusion

Hysteroscopic uterine evacuation of EPL by using TRD seems to be a safe and feasible procedure in selected cases, such as patients with recent uterine surgery or recurrent pregnancy losses. The hysteroscopic approach allows to precisely localise the insertion of the gestational sac inside the uterine cavity and to evaluate possible contributing complications with implantation (uterine malformations, adenomyosis, endometritis) in patients with recurrent pregnancy loss. Moreover, the endoscopic approach allows the clinician to perform hysteroembryoscopy prior to uterine evacuation and this technique seems to be more accurate than D&C for fetal chromosome karyotyping, with lower maternal cell contamination (Cholkeri-Singh et al., 2020).
